# A Population‐Based Study of Infectious Diseases Mortality Risk in Patients With Hematologic Malignancies 2000–2020

**DOI:** 10.1002/cam4.70850

**Published:** 2025-04-01

**Authors:** Wenshuai Zheng, Shenyu Wang, Bo Peng, Xiaoning Gao

**Affiliations:** ^1^ Department of Hematology Hainan Hospital of Chinese PLA General Hospital Sanya Hainan China; ^2^ Senior Department of Hematology Fifth Medical Center of Chinese PLA General Hospital Beijing China

**Keywords:** hematologic malignancies, infectious diseases mortality, population‐based, risk

## Abstract

**Background:**

Patients with hematologic malignancies are at high risk of dying from infectious diseases. However, little attention has been paid to infectious diseases mortality (IDM) in these patients. The aim of our study is to determine the incidence and trends of IDM in patients with hematologic malignancies, identify risk factors associated with IDM, and compare the risk of IDM in patients with the general United States population.

**Methods:**

The data of patients with hematologic malignancies between 2000 and 2020 was retrieved from the Surveillance, Epidemiology, and End Results program. Standardized mortality ratios (SMRs) and IDM rates were calculated. A competing risk model was performed to identify potential risk factors of IDM.

**Results:**

Among 700,678 patients, 15,028 IDM were identified with an IDM rate of 401.31/100,000 person‐years. Compared with the general population, the SMR of IDM was 3.34, and the elevated risk of IDM ran through the follow‐up period. For all cancer subtypes, the IDM rates were highest in the first 2 months after diagnosis and gradually declined thereafter. For all patients, the early period of diagnosis, older age, male, non‐Hispanic black, single or divorced/separated/widowed status, no chemotherapy, and no radiation were risk factors for IDM. For patients with Hodgkin lymphoma or non‐Hodgkin lymphoma, advanced stage was also a risk factor for IDM.

**Conclusion:**

Given the high risk of IDM in patients with hematologic malignancies, it is extremely important to identify patients at high risk of IDM and provide timely intervention to prevent early death from infections and improve prognosis.

## Graphical abstract

For hematologic malignancies, the infectious diseases mortality (IDM) rates were highest in the first 2 months after diagnosis and gradually declined thereafter. It is extremely important to identify patients at high risk of IDM and provide timely intervention to prevent early death from infections and improve prognosis.

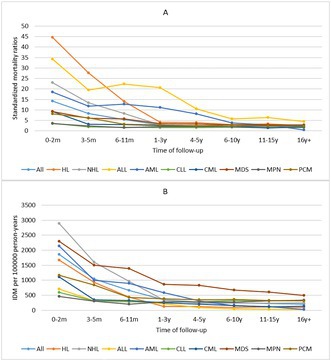

## 1 Introduction

In the United States (US), hematologic malignancies are a major public health problem and are an important cause of death. In 2023, there were an estimated 184,270 new patients with hematologic malignancies with 57,380 deaths in the US [1]. With the improvement in screening, diagnosis, treatment, and medical care, the mortality from primary hematologic malignancies has recently been decreasing, especially for chronic myeloid and lymphocytic leukemia (CML and CLL), Hodgkin lymphoma (HL)and non‐Hodgkin lymphoma (NHL) and plasma cell myeloma (PCM) [2, 3, 4, 5, 6], and non‐cancer deaths are gradually playing a dominant role in long‐term survival.

Due to more likely baseline immunodeficiencies and more often myelosuppressive treatment regimens, hematologic malignancies are usually associated with a higher risk of infection compared to solid malignancies [7, 8], which represents a serious cause of morbidity and mortality [9, 10, 11, 12], Considering the susceptibility of patients with hematologic malignancies to infection, infectious disease may be a key factor threatening long‐term survival. However, the infectious diseases mortality (IDM) in patients with hematologic malignancies has been rarely described in previous studies. The underestimation of the risk of IDM in patients with hematologic malignancies may result in missed opportunities for early intervention or less aggressive treatment modalities. Therefore, we conducted this nationwide population‐based study to provide a more comprehensive understanding of IDM in patients with hematologic malignancies.

## 2 Patients and Methods

### 2.1 Patients

Data of this study were obtained from the Surveillance, Epidemiology, and End Results (SEER) database of the National cancer Institute, which is an authoritative source of high‐quality cancer registries worldwide and currently covers approximately ~30% of the US population. It is freely available to the public and does not require informed consent from patients or institutional review board approval [13, 14]. Our analyses were limited to the SEER 17 registries database, published in April 2023 based on November 2022 submission.

Only hematologic malignancies were collected from the SEER database using International Classification of Diseases for Oncology 3rd edition (ICD‐O‐3) codes, 9590–9992. The exclusion criteria were (1) diagnosis confirmed only by autopsy or death certificate, (2) unknown age, and (3) patients who were not the primary malignancy, as Any correlation between a specific cancer and IDM would be complicated in patients with multiple primary cancers. According to histology, we further classified hematologic malignancies including: (1) HL (ICDO‐3 codes: 9650–9667); (2) NHL (ICD‐O‐3 codes: 9590–9597, 9670–9729, 9735–9738); (3) acute lymphocytic leukemia (ALL) (ICD‐O‐3 codes: 9811–9818, 9826, 9827, 9835–9837); (4) acute myeloid leukemia (AML) (ICD‐O‐3 codes: 9840, 9861, 9865, 9867, 9869, 9871–9874, 9891–9897, 9910–9911); (5) CLL (ICD‐O‐3 codes: 9823); (6) CML (ICD‐O‐3 codes: 9863, 9875, 9876, 9945, 9946); (7) myelodysplastic syndrome (MDS) (ICD‐O‐3 codes: 9980–9992); (8) myeloproliferative neoplasms (MPN) (ICD‐O‐3 codes: 9950–9962); (9) PCM (ICD‐O‐3 codes: 9731–9732, 9734); and (10) other (ICD‐O‐3 codes: 9733, 9740–9809, 9820, 9831–9834, 9860, 9866, 9870, 9898, 9920–9940, 9948, 9963–9975).

Causes of death were defined using the International Classification of Diseases 10 codes of National cancer for Health Statistics. IDM include the following five causes of death from the SEER database: pneumonia and influenza, septicemia, syphilis, tuberculosis, and other infectious and parasitic diseases including HIV.

Study variables included demographic information (age, gender, race, marital status), primary treatment modality (chemotherapy, radiation), year of diagnosis, survival time, vital status, and causes of death. Age at diagnosis was divided into five age groups: 0–19, 20–39, 40–59, 60–79, and ≥ 80 years. Race was classified into three groups: non‐Hispanic white (NHW), non‐Hispanic black (NHB) and other (non‐Hispanic American Indian/Alaska Native, non‐Hispanic Asian or Pacific Islande and Hispanic). Marital status was divided into four groups: married (including common law), single (never married), divorced, separated, and widowed (D/S/W), and other (Unmarried or Domestic Partner, Uknown). Chemotherapy and radiation were classified as yes and no/unknown. For patients with HL or NHL, summary stage for lymphoma was used to determine the exact stage. The stage was categorized into early stage (localized and regional), advanced stage (distant) and unknown. The year of diagnosis was divided into seven periods: 2000–2002, 2003–2005, 2006–2008, 2009–2011, 2012–2014, 2015–2017, and 2018–2020.

### 2.2 Statistical Analyses

Person‐years (PY) of follow‐up were accumulated from the initial cancer diagnosis to the first of the following dates: date of death, date lost to follow‐up, or date of study end (December 31, 2020). IDM rates were calculated as the number of IDM divided by PY of follow‐up. The standardized mortality ratios (SMRs) were calculated in SEER*Stat software using the MP‐SIR section, which compares the relative risk of IDM between patients with hematologic malignancies and the general US population, adjusted by age, race, and gender over the same time. The reference cohort was US mortality as reported in the National Vital Statistics System and maintained by the National Center for Health statistics. SMRs were calculated as the ratio of observed‐to‐expected deaths, and the 95% confidence interval (CI) was calculated using the exact method implemented in the SEER*Stat software.

To prevent bias resulting from varying intervals of follow‐up time for individuals diagnosed during different periods, we limited the follow‐up interval in the trend analyses of the year of diagnosis. For example, patients diagnosed in 2000–2002 were tracked until 2005 (an additional 3‐year minimum duration) and those diagnosed in 2015–2017 were tracked until 2020. All deaths during follow‐up were recorded. Due to inadequate follow‐up, patients diagnosed in 2018–2020 were not included in trend analyses.

Competing risk model was performed in the univariate and multivariate analyses to identify independent risk factors associated with IDM, in which death from other reasons was the competing event. Univariate analyses were performed using Gray's test, and factors with *p* < 0.1 in the univariate analyses were then included in the Fine‐Gray subdistribution hazard model, in which hazard ratio (HR) and 95% CI of each risk factor were calculated [15].

All statistical analyses were performed by the Surveillance Research Program, National Cancer Institute SEER*Stat software (seer.cancer.gov/seerstat) version 4.2.3 and R Statistical Software (R Foundation for Statistical Computing). All statistical analyses were two‐sided with a threshold of significance of *p* < 0.05.

## 3 Results

### 3.1 Patients' Characteristics

Finally, 700,678 patients with hematologic malignancies diagnosed from 2000 to 2020 were enrolled, including 40,923 (5.8%) HL, 261,103 (37.3%) NHL, 28,793 (4.1%) ALL, 45,725 (6.5%) AML, 74,039 (10.6%) CLL, 25,027 (3.6%) CML, 53,653 (7.7%) MDS, 43,647 (6.2%) MPN, and 92,478 (13.2%) PCM. The majority of patients were 60–79 years old (312,644, 44.6%), male (385,874, 55.1%), NHW (491,126, 70.1%) and married (358,696, 51.2%). Table 1 displays the baseline characteristics of all patients and those who died from infectious diseases.

**TABLE 1:** Incidence and SMRs of infectious diseases mortality in patients with hematologic malignancies by demographic and clinical characteristics.

Variable	Patients with hematologic malignancies No.	IDM No.	Person‐years	IDM per 100,000 Person‐years	SMRs	95% CI
Totall	700,678	15,028	3,744,751	401.31	3.34*	3.29–3.39
Period of diagnosis, years						
2000–2002	81,869	1659	240,004	691.24	5.13*	4.88–5.38
2003–2005	91,613	1681	281,651	596.84	4.37*	4.16–4.58
2006–2008	97,307	1663	305,879	543.68	4.30*	4.09–4.51
2009–2011	102,614	1763	328,841	536.13	4.33*	4.13–4.54
2012–2014	106,783	1646	343,795	478.77	4.08*	3.89–4.28
2015–2017	111,701	1611	359,714	447.86	4.13*	3.93–4.34
Age at diagnosis, years						
0–19	34,681	149	290,454	51.30	35.81*	30.3–42.05
20–39	58,517	1418	452,059	313.68	34.72*	32.93–36.57
40–59	173,128	4489	1,204,544	372.67	9.25*	8.98–9.52
60–79	312,644	5886	1,493,993	393.98	2.63*	2.57–2.7
≥ 80	121,708	3086	303,700	1016.13	1.78*	1.72–1.84
Time since diagnosis, months						
< 3	700,678	3021	162,080	1863.89	14.14*	13.64–14.65
3–5	622,875	1542	148,724	1036.82	8.30*	7.89–8.73
6–11	580,338	1814	272,827	664.89	5.44*	5.2–5.7
12–35	521,803	3048	883,782	344.88	2.85*	2.75–2.96
36–59	380,619	1789	662,657	269.97	2.25*	2.14–2.35
60–119	287,883	2502	1,023,849	244.37	2.04*	1.97–2.13
120–179	137,735	1034	455,136	227.18	1.94*	1.82–2.06
≥ 180	52,607	278	135,696	204.87	1.90*	1.68–2.14
Gender						
Female	314,804	5258	1,721,453	305.44	2.59*	2.52–2.66
Male	385,874	9770	2,023,297	482.88	3.96*	3.88–4.04
Race						
NHW	491,126	9436	2,686,931	351.18	2.67*	2.62–2.72
NHB	66,926	2713	331,986	817.20	6.72*	6.47–6.97
Other	142,626	2879	725,833	396.65	5.13*	4.94–5.32
Marital statuses						
Married	358,696	5986	1,990,567	300.72	2.48*	2.41–2.54
Single	138,684	4223	846,848	498.67	11.40*	11.05–11.74
D/S/W	147,937	3579	596,535	599.96	2.82*	2.73–2.92
Other	55,361	1240	310,800	398.97	2.78*	2.63–2.94
Chemotherapy						
No/Unknown	325,808	7867	1,641,332	479.31	2.98*	2.92–3.05
Yes	374,870	7161	2,103,419	340.45	3.84*	3.76–3.93
Radiation						
No/Unknown	619,358	13,539	3,187,615	424.74	3.37*	3.32–3.43
Yes	81,320	1489	557,135	267.26	3.06*	2.91–3.22
Dignosis						
HL	40,923	666	339,115	196.39	6.18*	5.72–6.67
NHL	261,103	7091	1,530,924	463.18	3.70*	3.61–3.78
ALL	28,793	245	182,844	133.99	14.95*	13.14–16.95
AML	45,725	586	105,237	556.84	10.08*	9.28–10.94
CLL	74,039	1383	450,894	306.72	1.76*	1.67–1.86
CML	25,027	332	136,313	243.56	2.70*	2.42–3.01
MDS	53,653	1803	182,621	987.29	4.06*	3.87–4.25
MPN	43,647	793	280,315	282.90	2.13*	1.98–2.28
PCM	92,478	1594	362,485	439.74	3.27*	3.11–3.44
Other	35,290	535	174,003	307.47	2.83*	2.59–3.08
Types of infectious diseases						
Tuberculosis	700,678	65	3,744,751	1.74	3.23*	2.49–4.11
Sypilis	700,678	2	3,744,751	0.05	1.55	0.19–5.59
Septicemia	700,678	3489	3,744,751	93.17	2.50*	2.41–2.58
Other infections and parasitic diseases including HIV	700,678	6739	3,744,751	179.96	8.69*	8.49–8.90
Pneumonia and influenza	700,678	4733	3,744,751	126.39	2.05*	2.00–2.11

Abbreviations: ALL, acute lymphocytic leukemia; AML, acute myeloid leukemia; CLL, chronic lymphocytic leukemia; CML, chronic myeloid leukemia; D/S/W, separated/divorced/widowed; HL, Hodgkin lymphoma; IDM, infectious diseases mortality; MDS, myelodysplastic syndrome; MPN, myeloproliferative neoplasms; NHB, non‐Hispanic black; NHL, non‐Hodgkin lymphoma; NHW, non‐Hispanic white; PCM, plasma cell myeloma; SMRs, standardized mortality ratios.

* *p* < 0.05.

### 3.2 Infectious Disease Mortality by Cancer Subtypes

**FIGURE 1:**
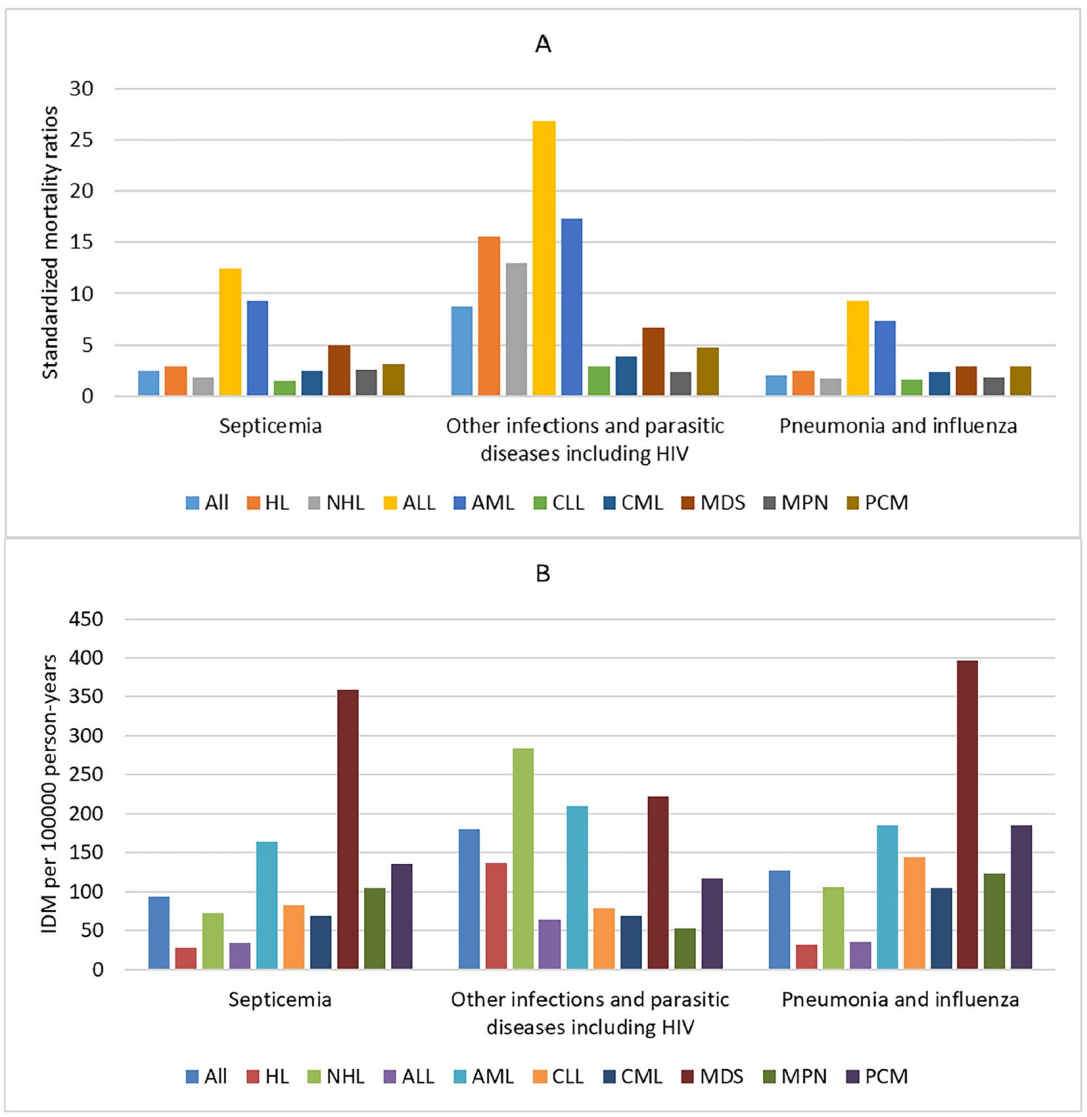
Standardized mortality ratios (SMRs) and infectious diseases mortality (IDM) rates per 100,000 person‐years according to cancer subtypes by infectious disease types. (Estimates of SMRs, IDM rates and all confidence intervals of SMRs can be found at corresponding supplementary table). X ax denotes the infectious disease types. Y ax denotes SMRs (A) and IDM rates (B) by infectious disease types. Color code denotes different cancer subtypes.

A total of 15,028 IDM was identified among all patients, giving an IDM rate of 401.31/100,000 PY. Of all IDM, 6739 (44.8%) were other infections and parasitic diseases including HIV with an IDM rate of 179.96/100,000 PY, followed by pneumonia and influenza (4733; 31.5%; IDM rate, 126.39/100,000 PY), and septicemia (3489; 23.2%; IDM rate, 97.13/100,000 PY). Among all cancer subtypes, MDS had the highest IDM rate of 987.29/100,000 PY, followed by AML (IDM rate, 556.84/100,000 PY) and NHL (IDM rate, 463.18/100,000 PY), while ALL had the lowest IDM rate of 133.99/100,000 PY followed by HL (IDM rate, 196.39/100,000 PY) and CML (IDM rate, 243.56/100,000 PY). The SMRs for all patients was 3.34 compared with the general US population, and the elevated risk of IDM continued through the follow‐up period. When considering cancer subtypes, the risk of IDM was greater than that of the general US population across all cancer subtypes. ALL had the highest SMRs of 14.95, followed by AML (SMRs, 10.08) and HL (SMRs, 6.18), while CLL had the lowest SMRs of 1.76, followed by MPN (SMRs, 2.13) and CML (SMRs, 2.70). The SMRs and IDM rates of different cancer subtypes according to different infectious diseases are seen in Figure 1.

### 3.3 Infectious Disease Mortality by Period of Diagnosis and Age at Diagnosis

**FIGURE 2:**
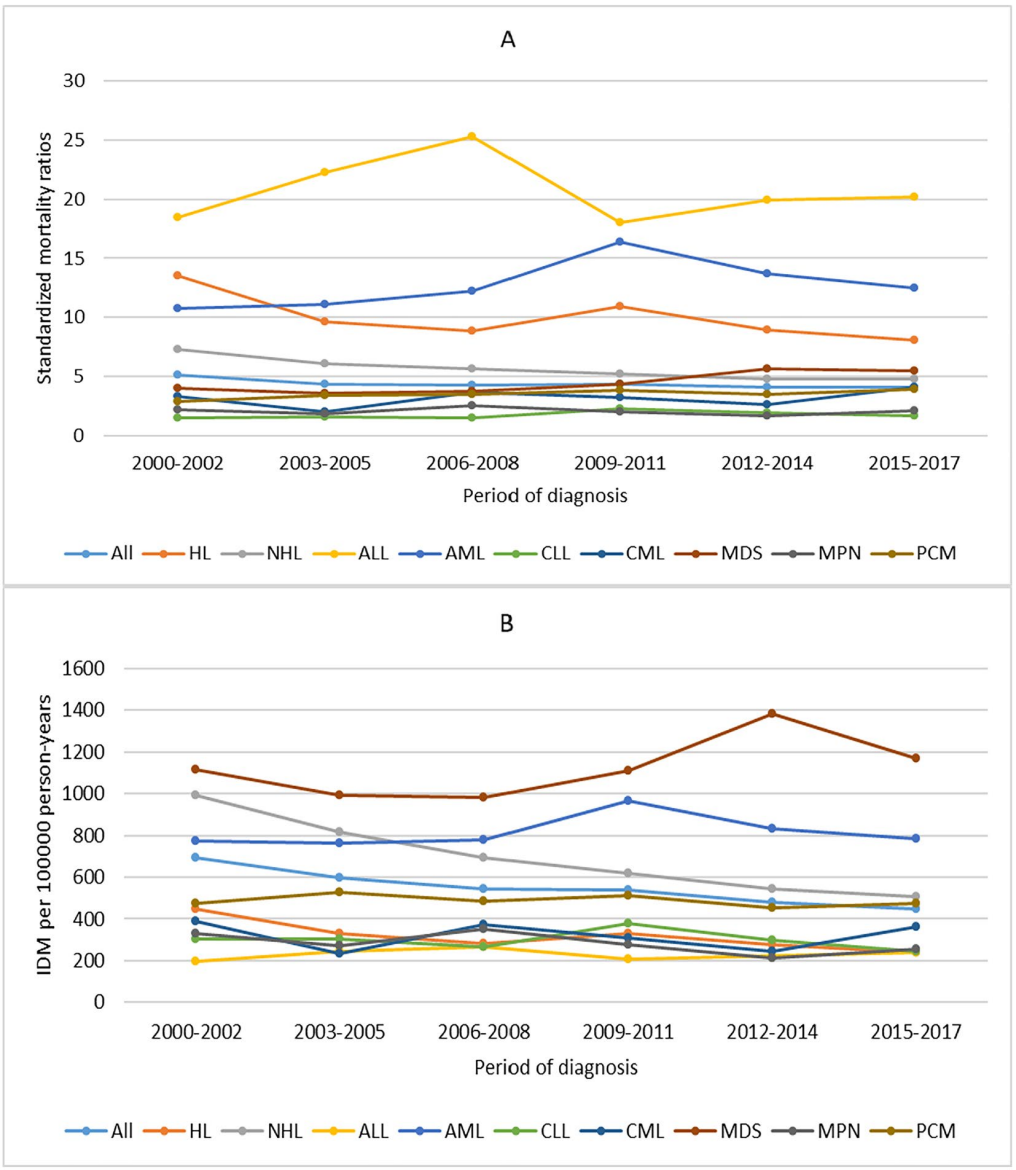
Standardized mortality ratios (SMRs) and infectious disease mortality (IDM) rates per 100,000 person‐years according to cancer subtypes by period of diagnosis. (Estimates of SMRs, IDM rates and all confidence intervals of SMRs can be found at corresponding supplementary table). X ax denotes the period of diagnosis. Y ax denotes SMRs (A) and IDM rates (B) by different periods of diagnosis. Color code denotes different cancer subtypes.

**FIGURE 3:**
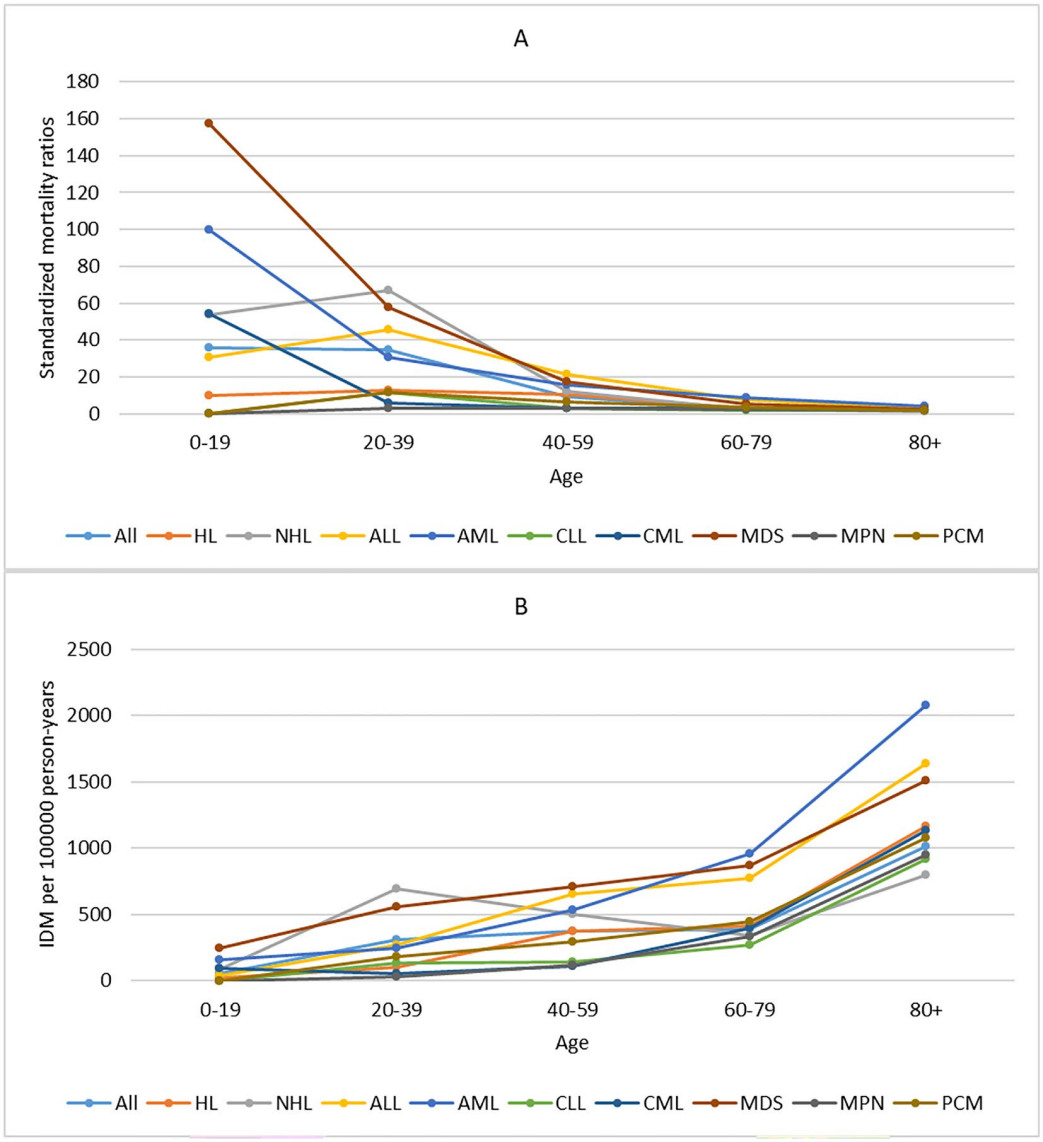
Standardized mortality ratios (SMRs) and infectious diseases mortality (IDM) rates per 100,000 person‐years according to cancer subtypes by age at diagnosis. (Estimates of SMRs, IDM rates and all confidence intervals of SMRs can be found at corresponding supplementary table). X ax denotes the age at diagnosis. Y ax denotes SMRs (A) and IDM rates (B) by different ages at diagnosis. Color code denotes different cancer subtypes.

For all patients, IDM rates showed a slow downward trend over the period of diagnosis. Patients diagnosed in 2014–2017 had the lowest IDM rate of 447.86/100,000 PY. The SMRs remained relatively stable over the period of diagnosis. We also observed a similar trend in patients with HL or NHL. For other cancer subtypes, the IDM rates and SMRs remained relatively stable over the period of diagnosis (Figure 2) When considering age at diagnosis, IDM rates increased with age, while SMRs decreased with age. Patients ≥ 80 years had the highest IDM rate of 1016.13/100,000 PY and the lowest SMRs of 1.78. The similar trend of IDM and SMRs with age at diagnosis was also observed in all cancer subtypes (Figure 3).

### 3.4 Infectious Disease Mortality Over Follow‐Up Time

**FIGURE 4:**
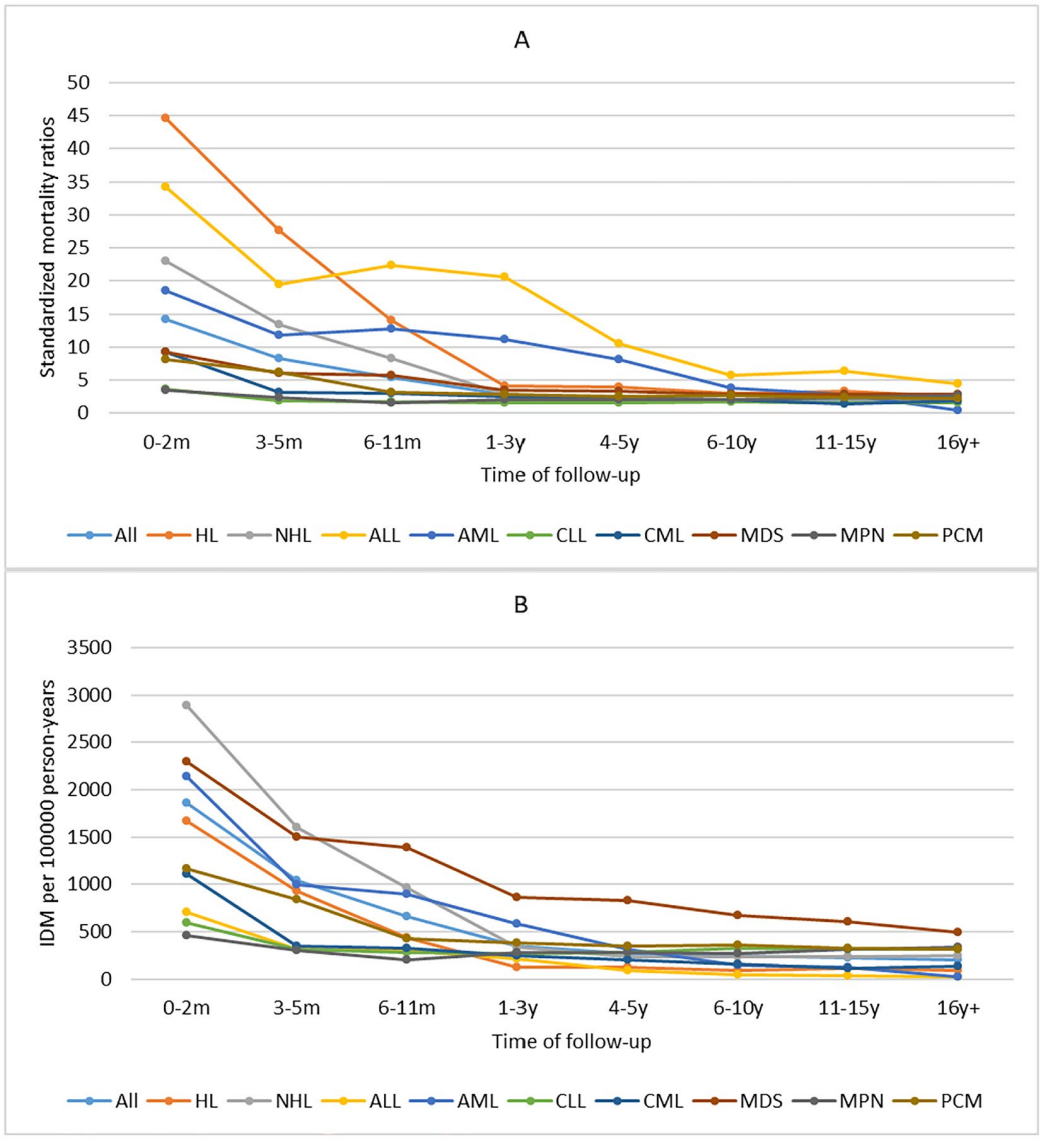
Standardized mortality ratios (SMRs) and infectious diseases mortality (IDM) rates per 100,000 person‐years according to cancer subtypes over follow‐up time. (Estimates of SMRs, IDM rates and all confidence intervals of SMRs can be found at corresponding supplementary table). X ax denotes the time after cancer diagnosis. Y ax denotes SMRs (A) and IDM rates (B) over follow‐up time. Color code denotes different cancer subtypes.

For all patients, the first 2 months after diagnosis represent the period with the highest IDM rate of 1863.89/100,000 PY, and IDM rates gradually declined thereafter. This trend was also seen across all cancer subtypes. Interestingly, the IDM rates of patients with AML, ALL, or MDS were highest in the first 2 months after diagnosis, remained stable between 3 months and 1 year after diagnosis, and gradually declined thereafter. The SMRs of all patients were also highest in the first 2 months after diagnosis (SMR, 14.14) and gradually declined thereafter, but compared with the general US population, the SMRs remained elevated throughout follow‐up time. This trend was also observed across all cancer subtypes. The SMRs trend in patients with AML, ALL, or MDS was similar to the trend of their IDM rates. (Figure 4).

### 3.5 Infectious Disease Mortality by Sex, Race, and Marital Status

**FIGURE 5:**
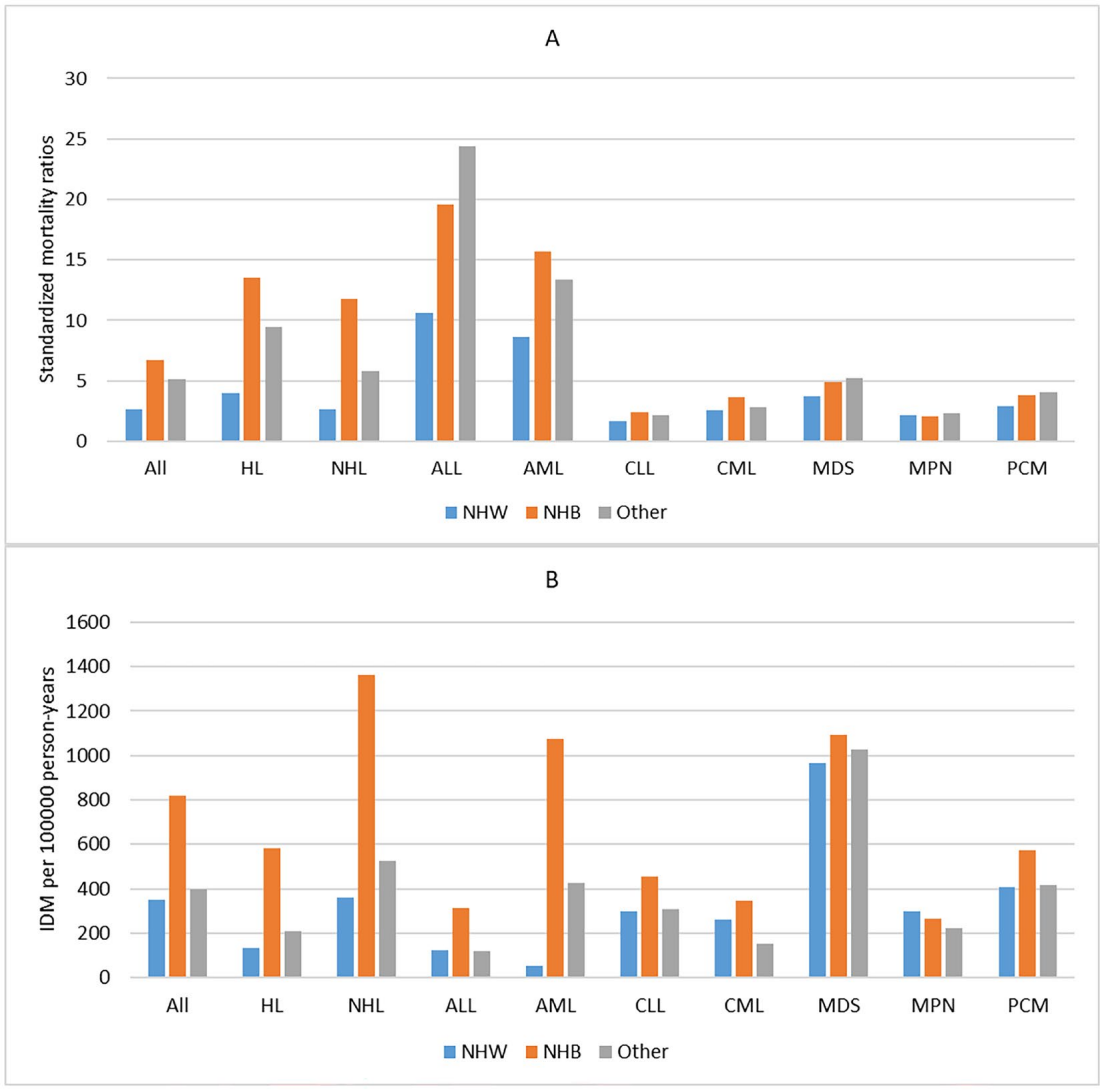
Standardized mortality ratios (SMRs) and infectious diseases mortality (IDM) rates per 100,000 person‐years according to cancer subtypes by races. (Estimates of SMRs, IDM rates and all confidence intervals of SMRs can be found at corresponding supplementary table). X ax denotes the cancer subtypes. Y ax denotes SMRs (A) and IDM rates (B) by different cancer subtypes. Color code denotes different races.

**FIGURE 6:**
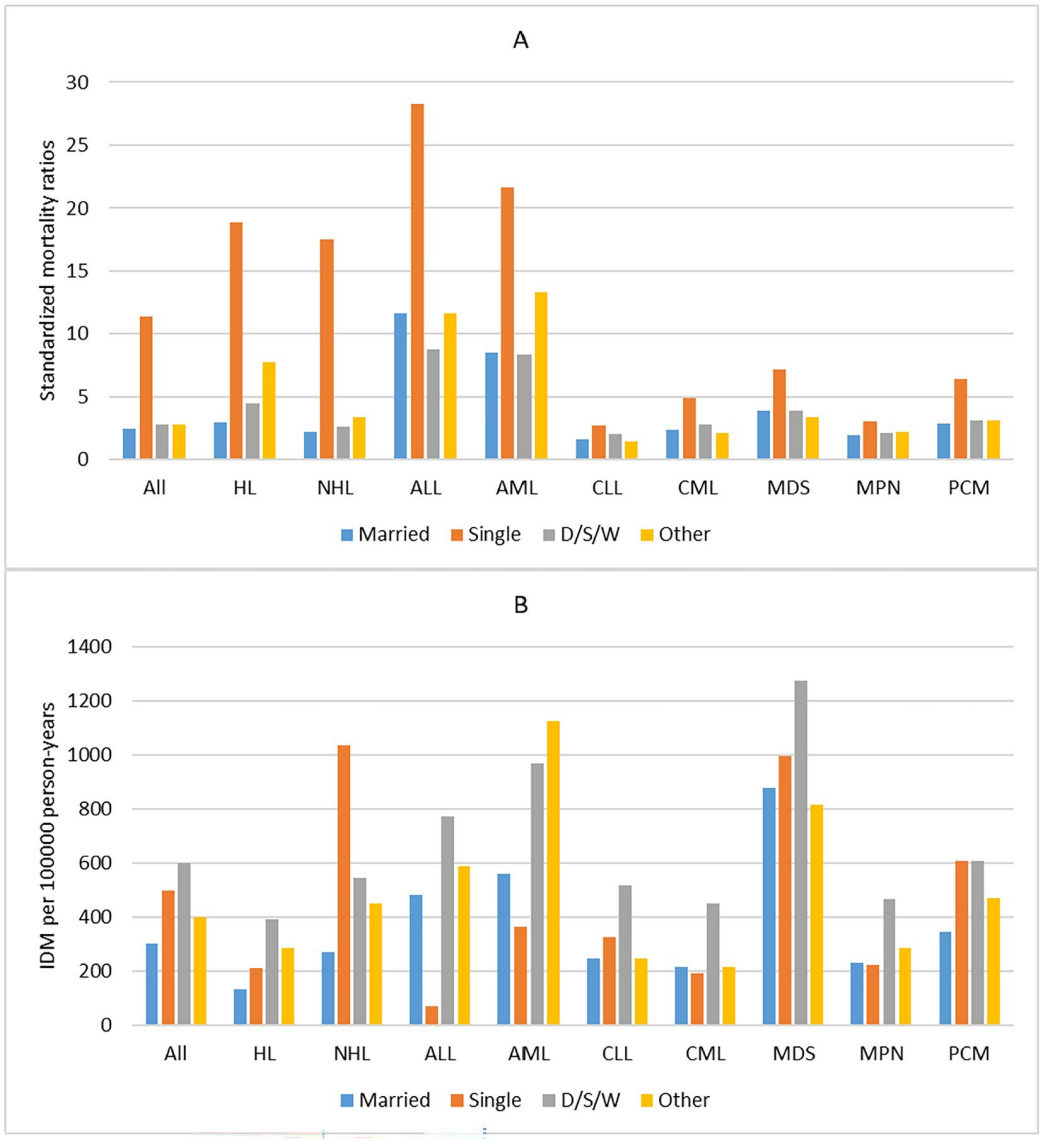
Standardized mortality ratios (SMRs) and infectious diseases mortality (IDM) rates per 100,000 person‐years according to cancer subtypes by marital status. (Estimates of SMRs, IDM rates and all confidence intervals of SMRs can be found at corresponding supplementary table). X ax denotes the marital status. Y ax denotes SMRs (A) and IDM rates (B) by different cancer subtypes. Color code denotes different marital statuses.

In our population, patients who died from infectious diseases were predominantly male (9770, 65.0%). For all patients, men had a higher IDM rate of 482.88/100,000 PY and SMRs of 3.96 than women (IDM rate, 305.44/100,000 PY; SMRs, 2.59). This phenomenon was also seen across all cancer subtypes. (Table 1, Table S1–S9) For race, NHW patients had the lowest IDM rate of 351.18/100,000 PY and SMRs of 2.67, while NHB patients had the highest IDM rate of 817.30/100,000 PY and SMRs of 6.72. This phenomenon was seen in most cancer subtypes, except MDS, MPN, and PCM. (Figure 5) When considering marital statuses, married patients had the lowest IDM rate of 300.72/100,000 PY and SMRs of 2.48 in all patients, while single patients had the highest SMRs of 11.40 and D/S/W patients had the highest IDM rate of 599.96/100,000 PY. However, the association between IDM rates and marital statuses varied by cancer subtypes. (Figure 6).

### 3.6 Infectious Disease Mortality by Treatment and Stage

For chemotherapy, patients who received chemotherapy had a lower IDM rate of 340.45/100,000 PY and higher SMRs of 3.84 compared with patients who did not receive chemotherapy in all patients. (Table 1) When considering cancer subtypes, the association between IDM rates and chemotherapy was varied. (Table S1) For radiation, patients who received radiation had lower IDM rates of 267.26/100,000 PY and SMRs of 3.06 compared with patients who did not receive radiation in all patients and patients with HL or NHL. (Table 1; Tables S1 and S2) When considering stage, patients with early stage had lower IDM rates and SMRs compared with patients with advanced stage in patients with HL or NHL. (Tables S1 and S2).

### 3.7 Internal Comparisons of Infectious Disease Mortality

The results of competing risk analyses are presented in Table 2. For all patients, all variables were significantly associated with IDM in both univariate and multivariate competing risk analyses. In multivariate analyses, we observed that the risk of IDM gradually increased with age, while it decreased with the period of diagnosis. Additionally, MDS had a higher risk of IDM compared with other cancer subtypes. Male patients (HR = 1.70) had a higher risk of IDM compared with female patients. When considering treatment, patients who received chemotherapy (HR = 0.84) and patients who received radiation (HR = 0.82) had a lower risk of IDM. Compared with NHW patients, the risk of IDM was higher in NHB patients (HR = 2.04). Compared with married patients, single patients (HR = 2.40) and D/S/W patients (HR = 1.53) had a higher risk of IDM.

**TABLE 2:** Univariate and multivariate competing risk analyses of risk factors of infectious diseases mortality in all patients.

	Univariate		Multivariate	
Variable	HR (95% CI)	*p*	HR (95% CI)	*p*
Dignosis				
MDS	Reference		Reference	
AML	0.39 (0.35–0.43)	< 0.01	0.42 (0.38–0.46)	< 0.01
NHL	0.81 (0.77–0.86)	< 0.01	0.86 (0.81–0.90)	< 0.01
PCM	0.53 (0.50–0.57)	< 0.01	0.52 (0.49–0.56)	< 0.01
CLL	0.56 (0.53–0.60)	< 0.01	0.57 (0.53–0.61)	< 0.01
MPN	0.55 (0.51–0.60)	< 0.01	0.56 (0.51–0.61)	< 0.01
CML	0.41 (0.36–0.46)	< 0.01	0.40 (0.36–0.45)	< 0.01
HL	0.48 (0.44–0.53)	< 0.01	0.50 (0.45–0.55)	< 0.01
ALL	0.26 (0.23–0.30)	< 0.01	0.47 (0.41–0.55)	< 0.01
Other	0.47 (0.43–0.52)	< 0.01	0.50 (0.46–0.55)	< 0.01
Period of diagnosis, years				
2000–2004	Reference		Reference	
2005–2009	0.87 (0.84–0.91)	< 0.01	0.83 (0.80–0.87)	< 0.01
2010–2014	0.75 (0.72–0.78)	< 0.01	0.71 (0.68–0.74)	< 0.01
2015–2020	0.56 (0.54–0.59)	< 0.01	0.53 (0.50–0.55)	< 0.01
Age at diagnosis, years				
0–19	Reference		Reference	
20–39	5.75 (4.86–6.81)	< 0.01	6.86 (5.73–8.20)	< 0.01
40–59	6.04 (5.13–7.16)	< 0.01	8.07 (6.75–9.66)	< 0.01
60–79	4.45 (3.78–5.24)	< 0.01	6.75 (5.64–8.08)	< 0.01
≥ 80	5.91 (5.02–6.97)	< 0.01	8.81 (7.35–10.58)	< 0.01
Gender				
Female	Reference		Reference	
Male	1.53 (1.48–1.58)	< 0.01	1.70 (1.64–1.76)	< 0.01
Race				
NHW	Reference		Reference	
NHB	2.21 (2.11–2.30)	< 0.01	2.13 (2.04–2.23)	< 0.01
Other	1.11 (1.07–1.16)	< 0.01	1.23 (1.18–1.28)	< 0.01
Marital statuse				
Married	Reference		Reference	
Single	1.88 (1.81–1.96)	< 0.01	2.39 (2.30–2.49)	< 0.01
D/S/W	1.44 (1.38–1.51)	< 0.01	1.53 (1.46–1.60)	< 0.01
Other	1.36 (1.28–1.45)	< 0.01	1.37 (1.28–1.45)	< 0.01
Chemotherapy				
No/Unknown	Reference		Reference	
Yes	0.79 (0.77–0.82)	< 0.01	0.88 (0.84–0.91)	< 0.01
Radiation				
No/Unknown	Reference		Reference	
Yes	0.80 (0.76–0.85)	< 0.01	0.79 (0.75–0.83)	< 0.01

Abbreviations: CI, confidence interval; D/S/W, separated/divorced/widowed; HR, hazard ratio; NHB, non‐Hispanic black; NHW, non‐Hispanic white.

## 4 Discussion

Our population‐based study determined the incidence and trends of IDM in patients with hematologic malignancies in the United States. For all patients, the IDM rate was 401.31/100,000 PY, with other infections and parasitic diseases, including HIV, being the most common infectious disease type. Our data demonstrated that patients with hematologic malignancies in the US had a noticeably higher risk of IDM than the general US population over the last 20 years. In addition, we also performed analyses of patient characteristics associated with the risk of IDM, in which early period of diagnosis, older age, male, NHB, single or divorced/separated/widowed status, no chemotherapy, and no radiation were risk factors for IDM.

Our study revealed that IDM rates and SMRs were highest in the first 2 months after diagnosis and then decreased over follow‐up time in all cancer subtypes. The possible reason is that patients at diagnosis had deficiencies in innate and adaptive immunity [8], which increased the risk of infection. Furthermore, most patients will receive treatment at diagnosis, most commonly chemotherapy and radiation. Chemotherapy and radiation can predispose patients to infection in a variety of ways, including bone marrow suppression and neutropenia, local tissue inflammation and breakdown, and lymphocyte depletion [16, 17, 18]. In addition, in the middle and later stages of the disease, the surviving patients often achieve remission and immune reconstitution, which would decrease the risk of infection. Interestingly, the IDM rates and SMRs of patients with AML, ALL, or MDS remained relatively stable between 3 months and 1 year after diagnosis and then gradually declined thereafter. For AML and ALL, this phenomenon may be explained by the continuously intensive chemotherapy regimen throughout the whole course, which places these patients at a constant highrisk ofneutropenia and infection [19, 20]. For MDS, the reason may be that the chemotherapy and continuously functional impairment of neutrophils increased the risk of infectious [21]. To our knowledge, we are the first to report that the Risk of IDM was highest in the first 2 months after diagnosis in all hematologic malignancies. Considering the characteristics of the risk of IDM over follow‐up time, we should pay more attention to the IDM risk and implement positive therapeutic intervention strategies against infection in patients with hematologic malignancies within 2 months after diagnosis.

In the multivariate competing risk analyses for all patients, patients diagnosed in a later period of diagnosis had a significantly lower risk of IDM compared with patients diagnosed in an early period of diagnosis. The IDM rates also showed a slow downward trend over the period of diagnosis. However, the SMRs were not decreased significantly over the period of diagnosis. The reason might be that SMRs are a relative comparison of the risk of IDM between patients and the general US population, while HR and IDM rate are internal comparisons of the risk of IDM in subgroups. This result indicated that there were no appreciable advancements in cancer treatment strategies associated with IDM, and the improved comprehensive care and the advanced progress of infection effectively reduced the risk of IDM in both patients with hematologic malignancies and the general US population. We also found that older patients are at higher risk of IDM compared with younger patients. The major explanation for this phenomenon is that the immune system's capacity to respond to infections declines with age [22]. Furthermore, older patients might be more vulnerable to the myelosuppressive effects of chemotherapy, which can lead to treatment‐related neutropenia, than younger patients [23]. In addition, older patients are typically frailer and have serious complications, which increase their risk of IDM [24]. In contrast to IMD rates, we found that patients diagnosed at a younger age had higher SMRs, and the SMRs continuously decreased with age. The main reason for this phenomenon is that infectious diseases are not prevalent in the younger general US population and are usually diagnosed in older general US population, and SMRs are a relative comparison of the risk of IDM between patients and the general US population.

In recent years, the role of sociodemographic factors in cancer is receiving increasing attention due to the advances in healthcare and the increased understanding of human health. The SEER database's social and demographic information has allowed us to discover previously unknown aspects of cancer. Multivariate competing risk analyses showed that male patients had a higher risk of IDM compared with female patients. Male patients also had higher IDM rates and SMRs than female patients in all patients and all cancer subtypes. This phenomenon may be attributed to the fundamental differences in the immune systems of male and female. Compared with male, female exhibit more vigorous innate, cell‐mediated, and humoral immune responses to antigenic challenges, which can reduce pathogen load and accelerate pathogen clearance [25]. Multivariate competing risk analyses showed that NHB patients had a higher risk of IDM compared with NHW patients. For all patients and most cancer subtypes, NHW patients also had the lowest IDM rates and SMRs, while NHB patients had the highest IDM rates and SMRs. This may be due to the fact that NHB patients had a higher likelihood of obesity and a lower likelihood of seeing a specialist, adhering to medication regimens, and receiving comprehensive follow‐up care due to higher cost [26, 27, 28]. Competing risk analyses showed that being single or D/S/W was also a risk factor for IDM compared with being married. Married patients also had the lowest IDM rates and SMRs in all patients and most cancer subtypes. This may be due to the fact that married patients can share their emotional burden, receive emotional and financial support from their spouses, and have better supportive care and adherence with prescribed treatments than unmarried patients [29]. Considering the impact of sociodemographic factors on IDM, the therapeutic intervention strategies against infections must take these factors into account to provide optimal disease management for patients with hematologic malignancies.

For most patients with hematologic malignancies, chemotherapy is the most common treatment measure. In our study, patients who received chemotherapy had a lower IDM rate compared with patients who did not receive chemotherapy in all patients and most cancer subtypes. Multivariate competing risk analyses also showed that chemotherapy was a protective factor for IDM. The possible explanation for this phenomenon is that most patients who received chemotherapy often get remission and reconstitution, which would decrease the risk of infection compared with patients who did not receive chemotherapy. However, the SMRs of patients who received chemotherapy were higher than that of patients who did not receive chemotherapy. This can be explained by the fact that most patients who received chemotherapy are young. In our study, 48.1% of patients who received chemotherapy were < 60 years old, while the portion in patients who did not receive chemotherapy was only 26.5%. As mentioned above, younger patients had higher SMRs compared with older patients. For patients with HL or NHL, radiation is also a treatment measure. In our study, patients who received radiation had a lower IDM rate and SMRs compared with patients who did not receive radiation in all patients and patients with HL or NHL. Multivariate competing risk analyses also showed that radiation was a protective factor for IDM. The reason for this result may be similar to the explanation for chemotherapy. For patients with HL or NHL, patients with early stage had a lower IDM rate and SMRs compared with patients with advanced stage. This phenomenon was rarely described in previous studies. The possible reason may be that patients with early stage usually had higher remission rates and better prognosis, which is more conducive to the recovery of immune function.

When considering each cancer subtype, our study showed that patients with MDS had the highest risk of IDM, followed by AML, NHL, PCM, and CLL, while patients with ALL had the lowest risk of IDM, followed by HL, CML, and MPN. As mentioned above, the high risk of IDM in patients with MDS, AML, and NHL can be explained by the continuously intensive chemotherapy regimen throughout the whole course and continuously functional impairment of neutrophil [19, 20, 21, 30]. For patients with PCM and CML, the high risk of IDM may be caused by the high proportion of older patients and immunodeficiency [31, 32]. The low risk of IDM in patients with HL, CML, and MPN was mainly contributed to by the weak chemotherapy regimen, which had little chance to induce neutropenia [33]. An interesting result was that patients with ALL had the lowest risk of IDM while they usually received an intensive chemotherapy regimen. This result was mainly caused by the high proportion of younger patients (59.2% patients younger than 20 years‐old), who had a low risk of IDM compared with older patients.

Due to the nature of the SEER registry, this study had several limitations. First, the retrospective nature of our study introduces inherent limitations and biases in terms of data collection and analyses. Second, the SEER database does not collect detailed therapeutic regimens of treatment. We could not evaluate the risk of IDM according to different chemotherapeutic agents. Third, we could not evaluate the risk of IDM of specific infections, such as HIV, which was included in the category of other infectious and parasitic diseases including HIV. Furthermore, the lack of individual‐level information, such as history of infectious diseases, hyperlipidemia, diabetes, and an unhealthy lifestyle, inevitably causes some bias to the research ending.

Despite these limitations, our study has several strengths. First, to our knowledge, the current study is the largest and most comprehensive characterization of IDM among hematologic malignancies using a national cancer registry with 20 years of data. Second, we examined the personal characteristics associated with the risk of IDM in more detail, especially sex, race, and marital status, which were underestimated in previous studies. These data underscore the importance of IDM for patients with hematologic malignancies and will assist oncologists in identifying patients who require positive infectious diseases support.

## Author Contributions

**Wenshuai Zheng:** conceptualization (equal), data curation (equal), formal analysis (equal), methodology (equal), software (equal), writing – original draft (equal). **Shenyu Wang:** data curation (equal), formal analysis (equal), methodology (equal). **Bo Peng:** formal analysis (equal), methodology (equal). **Xiaoning Gao:** conceptualization (equal), methodology (equal), supervision (equal), writing – review and editing (equal).

## Ethics Statement

The data of this study are obtained from the SEER database. The patient's data are public and anonymous, so this study does not require ethical approval and informed consent.

## Conflicts of Interest

The authors declare no conflicts of interest.

## Supporting information


Table S1‐S9.

## Data Availability

The data that support the findings of this study are openly available in the Surveillance, Epidemiology, and End Results (SEER) database of the National Cancer Institute at https://seer.cancer.gov/.
