# Supplementary material for: A Population‐Based Study of Infectious Diseases Mortality Risk in Patients With Hematologic Malignancies 2000–2020
Source: Cancer Med. 2025 Apr 1;14(7):e70850. doi: 10.1002/cam4.70850 (PMC11959415; doi:10.1002/cam4.70850)
Supplement: Supplementary file 1 — Table S1‐S9. [file CAM4-14-e70850-s001.docx]

Table S1 Incidence and SMRs of infectious diseases mortality in patients with hodgkin lymphoma by demographic and clinical characteristics

| Variable | Patients with  hematologic  malignancies No. | IDM  No. | Person-years | IDM per 100,000  Person-years | SMRs | 95% CI |
| --- | --- | --- | --- | --- | --- | --- |
| Period of diagnosis, years |  |  |  |  |  |  |
| 2000-2002 | 5,633 | 98 | 22,015 | 445.14 | 13.53^#^ | 10.98-16.49 |
| 2003-2005 | 5,896 | 76 | 23,034 | 329.90 | 9.62^#^ | 7.58-12.04 |
| 2006-2008 | 6,138 | 68 | 24,304 | 279.79 | 8.88^#^ | 6.90-11.26 |
| 2009-2011 | 6,033 | 79 | 23,772 | 332.32 | 10.90^#^ | 8.63-13.59 |
| 2012-2014 | 5,753 | 63 | 22,766 | 276.73 | 8.98^#^ | 6.90-11.48 |
| 2015-2017 | 5,850 | 54 | 22,702 | 237.86 | 8.06^#^ | 6.05-10.52 |
| Age at diagnosis, years |  |  |  |  |  |  |
| 0-19 | 5,493 | 10 | 162,555 | 18.54 | 10.11^#^ | 4.85-18.59 |
| 20-39 | 17,301 | 161 | 86,320 | 99.04 | 12.83^#^ | 10.93-14.98 |
| 40-59 | 10,284 | 320 | 33,039 | 370.72 | 10.24^#^ | 9.15-11.43 |
| 60-79 | 6,372 | 137 | 3,257 | 414.66 | 3.07^#^ | 2.58-3.63 |
| ≥ 80 | 1,473 | 38 | 3,257 | 1166.74 | 2.08^#^ | 1.47-2.85 |
| Time since diagnosis, months |  |  |  |  |  |  |
| < 3 | 40,923 | 166 | 9,924 | 1672.67 | 44.68^#^ | 38.14-52.02 |
| 3-5 | 38,970 | 89 | 9,520 | 934.84 | 27.72^#^ | 22.26-34.11 |
| 6-11 | 37,516 | 80 | 18,110 | 441.73 | 14.02^#^ | 11.12-17.45 |
| 12-35 | 35,380 | 82 | 64,907 | 126.33 | 4.12^#^ | 3.28-5.11 |
| 36-59 | 29,851 | 68 | 55,051 | 123.52 | 4.01^#^ | 3.11-5.08 |
| 60-119 | 25,326 | 97 | 102,087 | 95.02 | 3.05^#^ | 2.48-3.72 |
| 120-179 | 15,811 | 65 | 58,037 | 112.00 | 3.41^#^ | 2.63-4.34 |
| ≥180 | 7,570 | 19 | 21,478 | 88.46 | 2.59^#^ | 1.56-4.04 |
| Gender |  |  |  |  |  |  |
| Female | 18,510 | 149 | 181,235 | 94.38 | 3.42^#^ | 2.89-4.01 |
| Male | 22,413 | 517 | 157,879 | 285.26 | 8.06^#^ | 7.38-8.79 |
| Race |  |  |  |  |  |  |
| NHW | 26,711 | 305 | 231,918 | 131.51 | 4.00^#^ | 3.56-4.47 |
| NHB | 4,706 | 216 | 37,082 | 582.50 | 13.50^#^ | 11.76-15.42 |
| Other | 9,506 | 145 | 70,115 | 206.80 | 9.42^#^ | 7.95-11.08 |
| Marital status |  |  |  |  |  |  |
| Married | 16,761 | 185 | 140,151 | 132.00 | 2.99^#^ | 2.57-3.45 |
| Single | 18,176 | 339 | 159,025 | 213.17 | 18.83^#^ | 16.88-20.95 |
| D/S/W | 4,048 | 102 | 26,035 | 391.79 | 4.50^#^ | 3.67-5.46 |
| Other | 1,938 | 40 | 13,903 | 287.70 | 7.74^#^ | 5.53-10.54 |
| Chemotherapy |  |  |  |  |  |  |
| No/Unknown | 6,814 | 206 | 49,148 | 419.15 | 8.13^#^ | 7.06-9.32 |
| Yes | 34,109 | 460 | 289,967 | 158.64 | 5.58^#^ | 5.09-6.12 |
| Radiation |  |  |  |  |  |  |
| No/Unknown | 28,545 | 597 | 129,350 | 284.60 | 8.00^#^ | 7.37-8.67 |
| Yes | 12,378 | 69 | 209,765 | 53.34 | 2.09^#^ | 1.62-2.64 |
| Stage |  |  |  |  |  |  |
| Early stage | 22,741 | 190 | 208,881 | 90.96 | 3.21^#^ | 2.77-3.7 |
| Advanced stage | 16,229 | 447 | 114,058 | 391.90 | 10.63^#^ | 9.67-11.66 |
| Unknown | 1,953 | 29 | 16,175 | 179.29 | 4.43^#^ | 2.97-6.36 |
| Types of infectious disease |  |  |  |  |  |  |
| Septicemia | 40,923 | 96 | 339,115 | 28.31 | 2.9^#^ | 2.35-3.54 |
| Other infections and parasitic  diseases including HIV | 40,923 | 462 | 339,115 | 136.24 | 15.59^#^ | 14.2-17.08 |
| Pneumonia and influenza | 40,923 | 107 | 339,115 | 31.55 | 2.41^#^ | 1.98-2.91 |

IDM, infectious diseases mortality; SMRs, standardized mortality ratios; NHW, non-Hispanic white; NHB, non-Hispanic black; D/S/W, separated/divorced/widowed; # means *P* < 0.05.

Table S2 Incidence and SMRs of infectious diseases mortality in patients with non-hodgkin lymphoma by demographic and clinical characteristics

| Variable | Patients with  hematologic  malignancies No. | IDM  No. | Person-years | IDM per 100,000 Person-years | SMRs | 95% CI |
| --- | --- | --- | --- | --- | --- | --- |
| Period of diagnosis, years |  |  |  |  |  |  |
| 2000-2002 | 34,251 | 1,050 | 105,990 | 990.66 | 7.28^#^ | 6.85-7.74 |
| 2003-2005 | 36,285 | 949 | 116,332 | 815.77 | 6.08^#^ | 5.70-6.48 |
| 2006-2008 | 38,091 | 863 | 124,140 | 695.18 | 5.65^#^ | 5.28-6.04 |
| 2009-2011 | 37,284 | 769 | 123,940 | 620.46 | 5.22^#^ | 4.86-5.61 |
| 2012-2014 | 37,940 | 680 | 125,468 | 541.97 | 4.79^#^ | 4.44-5.16 |
| 2015-2017 | 39,236 | 647 | 128,381 | 503.97 | 4.78^#^ | 4.42-5.16 |
| Age at diagnosis, years |  |  |  |  |  |  |
| 0-19 | 4,793 | 37 | 43,043 | 85.96 | 53.75^#^ | 37.85-74.09 |
| 20-39 | 19,536 | 1,059 | 153,179 | 691.35 | 66.91^#^ | 62.94-71.06 |
| 40-59 | 74,236 | 2,832 | 563,193 | 502.85 | 12.50^#^ | 12.05-12.97 |
| 60-79 | 120,354 | 2,225 | 654,366 | 340.02 | 2.22^#^ | 2.13-2.32 |
| ≥ 80 | 42,184 | 938 | 117,143 | 800.73 | 1.39^#^ | 1.30-1.48 |
| Time since diagnosis, months |  |  |  |  |  |  |
| < 3 | 261,103 | 1,752 | 60,590 | 2,891.57 | 23.00^#^ | 21.94-24.1 |
| 3-5 | 232,803 | 889 | 55,561 | 1,600.04 | 13.46^#^ | 12.59-14.38 |
| 6-11 | 216,837 | 979 | 102,139 | 958.49 | 8.23^#^ | 7.72-8.76 |
| 12-35 | 195,905 | 1,160 | 339,843 | 341.33 | 2.89^#^ | 2.73-3.07 |
| 36-59 | 150,225 | 642 | 267,570 | 239.94 | 1.96^#^ | 1.81-2.11 |
| 60-119 | 118,687 | 1,027 | 437,705 | 234.63 | 1.81^#^ | 1.71-1.93 |
| 120-179 | 61,247 | 488 | 205,540 | 237.42 | 1.75^#^ | 1.60-1.91 |
| ≥180 | 23,994 | 154 | 61,977 | 248.48 | 1.87^#^ | 1.59-2.19 |
| Gender |  |  |  |  |  |  |
| Female | 119,352 | 2,177 | 719,203 | 302.70 | 2.40^#^ | 2.30-2.50 |
| Male | 141,751 | 4,914 | 811,721 | 605.38 | 4.86^#^ | 4.73-5.00 |
| Race |  |  |  |  |  |  |
| NHW | 185,442 | 4,057 | 1,127,505 | 359.82 | 2.66^#^ | 2.58-2.74 |
| NHB | 19,824 | 1,489 | 109,119 | 1,364.57 | 11.75^#^ | 11.16-12.36 |
| Other | 55,837 | 1,545 | 294,301 | 524.97 | 5.81^#^ | 5.53-6.11 |
| Marital status |  |  |  |  |  |  |
| Married | 141,977 | 2,420 | 893,719.70 | 270.78 | 2.24^#^ | 2.15-2.33 |
| Single | 44,704 | 2,764 | 267,038.49 | 1,035.06 | 17.52^#^ | 16.87-18.18 |
| D/S/W | 55,581 | 1,389 | 255,502.40 | 543.63 | 2.64^#^ | 2.51-2.79 |
| Other | 18,841 | 518 | 114,663.50 | 451.76 | 3.39^#^ | 3.10-3.69 |
| Chemotherapy |  |  |  |  |  |  |
| No/Unknown | 109,041 | 3,073 | 611,694.28 | 502.38 | 3.42^#^ | 3.30-3.54 |
| Yes | 152,062 | 4,018 | 919,229.81 | 437.11 | 3.94^#^ | 3.82-4.07 |
| Radiation |  |  |  |  |  |  |
| No/Unknown | 218,382 | 6,054 | 1,226,787 | 493.48 | 3.88^#^ | 3.78-3.98 |
| Yes | 42,721 | 1,037 | 304,137 | 340.97 | 2.90^#^ | 2.72-3.08 |
| Stage |  |  |  |  |  |  |
| Early stage | 111,470 | 2,543 | 749,958 | 339.09 | 2.74^#^ | 2.63-2.85 |
| Advanced stage | 128,680 | 3,977 | 660,305 | 602.30 | 4.91^#^ | 4.75-5.06 |
| Unknown | 20,953 | 571 | 120,660 | 473.23 | 3.20^#^ | 2.94-3.47 |
| Types of infectious disease |  |  |  |  |  |  |
| Septicemia | 261,103 | 1104 | 1,530,924 | 72.11 | 1.86^#^ | 1.75-1.98 |
| Other infections and parasitic  diseases including HIV | 261,103 | 4337 | 1,530,924 | 283.29 | 13.02^#^ | 12.64-13.42 |
| Pneumonia and influenza | 261,103 | 1628 | 1,530,924 | 106.34 | 1.66^#^ | 1.58-1.74 |

IDM, infectious diseases mortality; SMRs, standardized mortality ratios; NHW, non-Hispanic white; NHB, non-Hispanic black; D/S/W, separated/divorced/widowed; # means *P* < 0.05.

Table S3 Incidence and SMRs of infectious diseases mortality in patients with acute lymphomatic leukemia by demographic and clinical characteristics

| Variable | Patients with  hematologic  malignancies No. | IDM  No. | Person-years | IDM per 100,000  Person-years | SMRs | 95% CI |
| --- | --- | --- | --- | --- | --- | --- |
| Period of diagnosis, years |  |  |  |  |  |  |
| 2000-2002 | 3,469 | 22 | 11,240 | 195.74 | 18.43^#^ | 11.55-27.90 |
| 2003-2005 | 3,635 | 29 | 11,914 | 243.41 | 22.21^#^ | 14.88-31.90 |
| 2006-2008 | 3,837 | 34 | 12,900 | 263.56 | 25.30^#^ | 17.52-35.35 |
| 2009-2011 | 4,215 | 29 | 14,126 | 205.30 | 18.01^#^ | 12.06-25.87 |
| 2012-2014 | 4,462 | 33 | 14,766 | 223.49 | 19.95^#^ | 13.73-28.01 |
| 2015-2017 | 4,645 | 37 | 15,407 | 240.14 | 20.21^#^ | 14.23-27.86 |
| Age at diagnosis, years |  |  |  |  |  |  |
| 0-19 | 17,055 | 53 | 144,559 | 36.66 | 30.76^#^ | 23.04-40.24 |
| 20-39 | 4,224 | 50 | 18,526 | 269.89 | 45.65^#^ | 33.88-60.18 |
| 40-59 | 3,834 | 85 | 13,054 | 651.13 | 21.54^#^ | 17.21-26.64 |
| 60-79 | 2,944 | 47 | 6,094 | 771.19 | 7.41^#^ | 5.44-9.85 |
| ≥ 80 | 736 | 10 | 612 | 1,635.19 | 3.05^#^ | 1.46-5.61 |
| Time since diagnosis, months |  |  |  |  |  |  |
| < 3 | 28,793 | 48 | 6,789 | 707.00 | 34.17^#^ | 25.20-45.31 |
| 3-5 | 26,352 | 20 | 6,385 | 313.22 | 19.51^#^ | 11.91-30.12 |
| 6-11 | 25,046 | 36 | 11,770 | 305.87 | 22.31^#^ | 15.63-30.89 |
| 12-35 | 22,442 | 81 | 37,915 | 213.64 | 20.60^#^ | 16.36-25.60 |
| 36-59 | 16,534 | 25 | 29,405 | 85.02 | 10.47^#^ | 6.78-15.46 |
| 60-119 | 13,138 | 21 | 50,942 | 41.22 | 5.81^#^ | 3.60-8.88 |
| 120-179 | 7,723 | 11 | 28,555 | 38.52 | 6.32^#^ | 3.16-11.32 |
| ≥180 | 3,844 | 3 | 11,084 | 27.07 | 4.5 | 0.93-13.15 |
| Gender |  |  |  |  |  |  |
| Female | 12,418 | 99 | 79,394 | 124.69 | 14.22^#^ | 11.56-17.32 |
| Male | 16,375 | 146 | 103,450 | 141.13 | 15.49^#^ | 13.08-18.22 |
| Race |  |  |  |  |  |  |
| NHW | 13,363 | 112 | 89,689 | 124.88 | 10.61^#^ | 8.73-12.76 |
| NHB | 2,161 | 37 | 11,859 | 312.00 | 19.55^#^ | 13.77-26.95 |
| Other | 13,269 | 96 | 81,296 | 118.09 | 24.42^#^ | 19.78-29.83 |
| Marital status |  |  |  |  |  |  |
| Married | 5,933 | 97 | 20,130 | 481.87 | 11.66^#^ | 9.45-14.22 |
| Single | 20,630 | 108 | 157,099 | 68.75 | 28.25^#^ | 23.17-34.11 |
| D/S/W | 1,644 | 29 | 3,749 | 773.53 | 8.80^#^ | 5.89-12.64 |
| Other | 586 | 11 | 1,867 | 589.31 | 11.65^#^ | 5.82-20.85 |
| Chemotherapy |  |  |  |  |  |  |
| No/Unknown | 2,077 | 37 | 5,618 | 658.58 | 9.20^#^ | 6.48-12.69 |
| Yes | 26,716 | 208 | 177,226 | 117.36 | 16.82^#^ | 14.62-19.27 |
| Types of infectious disease |  |  |  |  |  |  |
| Septicemia | 28,793 | 63 | 182,844 | 34.46 | 12.48^#^ | 9.59-15.96 |
| Other infections and parasitic  diseases including HIV | 28,793 | 116 | 182,844 | 63.44 | 26.87^#^ | 22.2-32.23 |
| Pneumonia and influenza | 28,793 | 64 | 182,844 | 35.00 | 9.26^#^ | 7.13-11.82 |

IDM, infectious diseases mortality; SMRs, standardized mortality ratios; NHW, non-Hispanic white; NHB, non-Hispanic black; D/S/W, separated/divorced/widowed; # means *P* < 0.05.

Table S4 Incidence and SMRs of infectious diseases mortality in patients with acute myeloid leukemia by demographic and clinical characteristics

| Variable | Patients with  hematologic  malignancies No. | IDM  No. | Person-years | IDM per  100,000  Person-years | SMRs | 95% CI |
| --- | --- | --- | --- | --- | --- | --- |
| Period of diagnosis, years |  |  |  |  |  |  |
| 2000-2002 | 6,552 | 64 | 8,258 | 774.96 | 10.79^#^ | 8.31-13.78 |
| 2003-2005 | 6,162 | 64 | 8,381 | 763.63 | 11.08^#^ | 8.53-14.14 |
| 2006-2008 | 6,370 | 71 | 9,093 | 780.80 | 12.22^#^ | 9.54-15.41 |
| 2009-2011 | 6,333 | 94 | 9,716 | 967.48 | 16.36^#^ | 13.22-20.03 |
| 2012-2014 | 6,629 | 85 | 10,189 | 834.23 | 13.72^#^ | 10.96-16.96 |
| 2015-2017 | 6,793 | 86 | 10,959 | 784.71 | 12.50^#^ | 10.00-15.43 |
| Age at diagnosis, years |  |  |  |  |  |  |
| 0-19 | 3,020 | 30 | 19,368 | 154.89 | 99.93^#^ | 67.43-142.66 |
| 20-39 | 4,360 | 52 | 21,272 | 244.45 | 30.63^#^ | 22.87-40.16 |
| 40-59 | 10,166 | 190 | 35,622 | 533.38 | 15.89^#^ | 13.71-18.32 |
| 60-79 | 19,260 | 245 | 25,653 | 955.07 | 8.91^#^ | 7.83-10.09 |
| ≥ 80 | 8,919 | 69 | 3,323 | 2,076.31 | 4.15^#^ | 3.23-5.25 |
| Time since diagnosis, months |  |  |  |  |  |  |
| < 3 | 45,725 | 185 | 8,639 | 2,141.53 | 18.46^#^ | 15.90-21.32 |
| 3-5 | 30,245 | 66 | 6,636 | 994.57 | 11.81^#^ | 9.13-15.02 |
| 6-11 | 24,969 | 93 | 10,381 | 895.86 | 12.77^#^ | 10.31-15.65 |
| 12-35 | 18,168 | 142 | 24,140 | 588.24 | 11.24^#^ | 9.47-13.25 |
| 36-59 | 8,940 | 48 | 15,079 | 318.32 | 8.11^#^ | 5.98-10.75 |
| 60-119 | 6,516 | 36 | 23,886 | 150.71 | 3.89^#^ | 2.72-5.39 |
| 120-179 | 3,466 | 15 | 12,158 | 123.37 | 2.80^#^ | 1.57-4.63 |
| ≥180 | 1,542 | 1 | 4,318 | 23.16 | 0.48 | 0.01-2.70 |
| Gender |  |  |  |  |  |  |
| Female | 20,737 | 234 | 50,358 | 464.67 | 9.94^#^ | 8.71-11.30 |
| Male | 24,988 | 352 | 54,879 | 641.41 | 10.18^#^ | 9.15-11.30 |
| Race |  |  |  |  |  |  |
| NHW | 31,338 | 37 | 68,103 | 54.33 | 8.62^#^ | 7.77-9.55 |
| NHB | 3,763 | 93 | 8,653 | 1,074.83 | 15.69^#^ | 12.67-19.23 |
| Other | 10,624 | 122 | 28,481 | 428.35 | 13.31^#^ | 11.05-15.89 |
| Marital status |  |  |  |  |  |  |
| Married | 23,757 | 299 | 53,107 | 563.01 | 8.49^#^ | 7.55-9.50 |
| Single | 9,819 | 135 | 36,988 | 364.99 | 21.63^#^ | 18.14-25.60 |
| D/S/W | 10,128 | 116 | 11,951 | 970.59 | 8.33^#^ | 6.88-9.99 |
| Other | 2,021 | 36 | 3,191 | 1,128.25 | 13.31^#^ | 9.33-18.43 |
| Chemotherapy |  |  |  |  |  |  |
| No/Unknown | 13,143 | 156 | 7,529 | 2,071.92 | 11.68^#^ | 9.92-13.67 |
| Yes | 32.582 | 430 | 97,708 | 440.09 | 9.61^#^ | 8.72-10.56 |
| Types of infectious disease |  |  |  |  |  |  |
| Septicemia | 45,725 | 172 | 105,237 | 163.44 | 9.31^#^ | 7.97-10.8 |
| Other infections and parasitic  diseases including HIV | 45,725 | 220 | 105,237 | 209.05 | 17.26^#^ | 15.06-19.7 |
| Pneumonia and influenza | 45,725 | 194 | 105,237 | 184.35 | 7.31^#^ | 6.32-8.42 |

IDM, infectious diseases mortality; SMRs, standardized mortality ratios; NHW, non-Hispanic white; NHB, non-Hispanic black; D/S/W, separated/divorced/widowed; # means *P* < 0.05.

Table S5 Incidence and SMRs of infectious diseases mortality in patients with chronic lymphomatic leukemia by demographic and clinical characteristics

| Variable | Patients with  hematologic  malignancies No. | IDM  No. | Person-years | IDM per  100,000  Person-years | SMRs | 95% CI |
| --- | --- | --- | --- | --- | --- | --- |
| Period of diagnosis, years |  |  |  |  |  |  |
| 2000-2002 | 7,750 | 86 | 28,245 | 304.48 | 1.50^#^ | 1.2-1.86 |
| 2003-2005 | 8,833 | 98 | 32,405 | 302.42 | 1.58^#^ | 1.28-1.92 |
| 2006-2008 | 9,206 | 92 | 34,717 | 265.00 | 1.51^#^ | 1.22-1.85 |
| 2009-2011 | 11,310 | 160 | 42,522 | 376.28 | 2.27^#^ | 1.93-2.65 |
| 2012-2014 | 12,221 | 139 | 46,591 | 298.34 | 1.93^#^ | 1.62-2.28 |
| 2015-2017 | 12,702 | 118 | 48,391 | 243.85 | 1.72^#^ | 1.42-2.06 |
| Age at diagnosis, years |  |  |  |  |  |  |
| 0-19 | 36 | 0 | 215 | - | - | ­- |
| 20-39 | 653 | 7 | 5,125 | 136.59 | 11.63^#^ | 4.68-23.97 |
| 40-59 | 16,522 | 185 | 131,812 | 140.35 | 3.15^#^ | 2.71-3.64 |
| 60-79 | 41,695 | 694 | 259,720 | 267.21 | 1.69^#^ | 1.57-1.83 |
| ≥ 80 | 15,133 | 497 | 54,021 | 920.00 | 1.58^#^ | 1.44-1.72 |
| Time since diagnosis, months |  |  |  |  |  |  |
| < 3 | 74,039 | 106 | 17,933 | 591.08 | 3.59^#^ | 2.94-4.34 |
| 3-5 | 70,443 | 55 | 17,234 | 319.14 | 1.96^#^ | 1.48-2.55 |
| 6-11 | 67,931 | 91 | 32,893 | 276.66 | 1.70^#^ | 1.37-2.09 |
| 12-35 | 64,027 | 307 | 112,909 | 271.90 | 1.65^#^ | 1.47-1.85 |
| 36-59 | 49,630 | 238 | 86,357 | 275.60 | 1.63^#^ | 1.43-1.85 |
| 60-119 | 37,338 | 407 | 126,091 | 322.78 | 1.80^#^ | 1.63-1.98 |
| 120-179 | 15,312 | 144 | 46,189 | 311.76 | 1.57^#^ | 1.32-1.85 |
| ≥180 | 4,766 | 35 | 11,288 | 310.07 | 1.51^#^ | 1.05-2.10 |
| Gender |  |  |  |  |  |  |
| Female | 30,094 | 528 | 186,650.44 | 282.88 | 1.60^#^ | 1.47-1.74 |
| Male | 43,945 | 855 | 264,243.31 | 323.57 | 1.88^#^ | 1.76-2.01 |
| Race |  |  |  |  |  |  |
| NHW | 62,478 | 1,147 | 387,226 | 296.21 | 1.69^#^ | 1.59-1.79 |
| NHB | 5,164 | 122 | 26,774 | 455.67 | 2.40^#^ | 1.99-2.86 |
| Other | 6,397 | 114 | 36,894 | 308.99 | 2.15^#^ | 1.77-2.58 |
| Marital status |  |  |  |  |  |  |
| Married | 39,415 | 633 | 254,439 | 248.78 | 1.59^#^ | 1.46-1.71 |
| Single | 7,889 | 143 | 43,908 | 325.68 | 2.70^#^ | 2.28-3.18 |
| D/S/W | 16,419 | 439 | 84,709 | 518.25 | 2.04^#^ | 1.86-2.25 |
| Other | 10,316 | 168 | 67,837 | 247.65 | 1.43^#^ | 1.22-1.67 |
| Chemotherapy |  |  |  |  |  |  |
| No/Unknown | 62,094 | 1,132 | 384,122 | 294.70 | 1.64^#^ | 1.55-1.74 |
| Yes | 11,945 | 251 | 66,772 | 375.90 | 2.62^#^ | 2.31-2.96 |
| Types of infectious disease |  |  |  |  |  |  |
| Septicemia | 74,039 | 371 | 450,894 | 82.28 | 1.52^#^ | 1.37-1.68 |
| Other infections and parasitic  diseases including HIV | 74,039 | 355 | 450,894 | 78.73 | 2.86^#^ | 2.57-3.17 |
| Pneumonia and influenza | 74,039 | 651 | 450,894 | 144.38 | 1.58^#^ | 1.46-1.7 |

IDM, infectious diseases mortality; SMRs, standardized mortality ratios; NHW, non-Hispanic white; NHB, non-Hispanic black; D/S/W, separated/divorced/widowed; # means *P* < 0.05.

Table S6 Incidence and SMRs of infectious diseases mortality in patients with chronic myeloid leukemia by demographic and clinical characteristics

| Variable | Patients with  hematologic  malignancies No. | IDM  No. | Person-years | IDM per 100,000 Person-years | SMRs | 95% CI |
| --- | --- | --- | --- | --- | --- | --- |
| Period of diagnosis, years |  |  |  |  |  |  |
| 2000-2002 | 2,976 | 33 | 8,457 | 390.20 | 3.30^#^ | 2.27-4.63 |
| 2003-2005 | 3,016 | 22 | 9,490 | 231.82 | 2.04^#^ | 1.28-3.09 |
| 2006-2008 | 3,271 | 39 | 10,535 | 370.20 | 3.66^#^ | 2.61-5.01 |
| 2009-2011 | 3,613 | 38 | 12,259 | 309.98 | 3.27^#^ | 2.32-4.49 |
| 2012-2014 | 3,868 | 32 | 13,208 | 242.28 | 2.60^#^ | 1.78-3.67 |
| 2015-2017 | 4,187 | 50 | 13,911 | 359.42 | 4.13^#^ | 3.07-5.45 |
| Age at diagnosis, years |  |  |  |  |  |  |
| 0-19 | 707 | 5 | 5,501 | 90.89 | 54.35^#^ | 17.65-126.83 |
| 20-39 | 3,547 | 15 | 27,732 | 54.09 | 5.74^#^ | 3.21-9.46 |
| 40-59 | 7,343 | 62 | 54,371 | 114.03 | 3.03^#^ | 2.32-3.88 |
| 60-79 | 9,499 | 162 | 40,976 | 395.35 | 2.88^#^ | 2.45-3.36 |
| ≥ 80 | 3,931 | 88 | 7,733 | 1,137.97 | 2.03^#^ | 1.63-2.50 |
| Time since diagnosis, months |  |  |  |  |  |  |
| < 3 | 25,027 | 65 | 5,883 | 1,104.94 | 9.28^#^ | 7.16-11.83 |
| 3-5 | 22,774 | 19 | 5,472 | 347.25 | 3.12^#^ | 1.88-4.87 |
| 6-11 | 21,404 | 33 | 10,119 | 326.12 | 3.05^#^ | 2.10-4.29 |
| 12-35 | 19,366 | 80 | 32,486 | 246.26 | 2.54^#^ | 2.01-3.16 |
| 36-59 | 13,867 | 49 | 24,001 | 204.16 | 2.35^#^ | 1.74-3.10 |
| 60-119 | 10,405 | 60 | 37,008 | 162.13 | 2.01^#^ | 1.54-2.59 |
| 120-179 | 4,979 | 19 | 16,344 | 116.25 | 1.47 | 0.89-2.30 |
| ≥180 | 1,875 | 7 | 5,001 | 139.97 | 1.83 | 0.74-3.77 |
| Gender |  |  |  |  |  |  |
| Female | 10,491 | 133 | 58,428 | 227.63 | 2.63^#^ | 2.20-3.12 |
| Male | 14,536 | 199 | 77,885 | 255.50 | 2.75^#^ | 2.38-3.16 |
| Race |  |  |  |  |  |  |
| NHW | 16,927 | 233 | 89,839 | 259.35 | 2.54^#^ | 2.23-2.89 |
| NHB | 2,551 | 50 | 14,364 | 348.10 | 3.66^#^ | 2.71-4.82 |
| Other | 5,549 | 49 | 32,111 | 152.60 | 2.81^#^ | 2.08-3.71 |
| Marital status |  |  |  |  |  |  |
| Married | 12,777 | 158 | 73,239 | 215.73 | 2.41^#^ | 2.05-2.82 |
| Single | 4,960 | 57 | 29,883 | 190.74 | 4.93^#^ | 3.73-6.39 |
| D/S/W | 4,944 | 87 | 19,313 | 450.47 | 2.76^#^ | 2.21-3.40 |
| Other | 2,346 | 30 | 13,878 | 216.17 | 2.11^#^ | 1.42-3.01 |
| Chemotherapy |  |  |  |  |  |  |
| No/Unknown | 7,738 | 170 | 31,321 | 542.76 | 3.58^#^ | 3.06-4.16 |
| Yes | 17,289 | 162 | 104,992 | 154.30 | 2.15^#^ | 1.83-2.51 |
| Types of infectious disease |  |  |  |  |  |  |
| Septicemia | 25,027 | 94 | 136,313 | 68.96 | 2.46^#^ | 1.99-3.01 |
| Other infections and parasitic  diseases including HIV | 25,027 | 142 | 136,313 | 104.17 | 2.37^#^ | 1.99-2.79 |
| Pneumonia and influenza | 25,027 | 93 | 136,313 | 68.23 | 3.87^#^ | 3.12-4.74 |

IDM, infectious diseases mortality; SMRs, standardized mortality ratios; NHW, non-Hispanic white; NHB, non-Hispanic black; D/S/W, separated/divorced/widowed; # means *P* < 0.05.

Table S7 Incidence and SMRs of infectious diseases mortality in patients with myelodysplastic syndrome by demographic and clinical characteristics

| Variable | Patients with  hematologic  malignancies No. | IDM  No. | Person-years | IDM per 100,000 Person-years | SMRs | 95% CI |
| --- | --- | --- | --- | --- | --- | --- |
| Period of diagnosis, years |  |  |  |  |  |  |
| 2000-2002 | 3,917 | 102 | 9,132 | 1,116.93 | 4.02^#^ | 3.27-4.88 |
| 2003-2005 | 7,221 | 184 | 18,536 | 992.68 | 3.59^#^ | 3.09-4.15 |
| 2006-2008 | 8,509 | 213 | 21,726 | 980.37 | 3.78^#^ | 3.29-4.32 |
| 2009-2011 | 9,471 | 269 | 24,265 | 1,108.57 | 4.37^#^ | 3.86-4.92 |
| 2012-2014 | 8,714 | 293 | 21,208 | 1,381.58 | 5.68^#^ | 5.05-6.37 |
| 2015-2017 | 8,218 | 230 | 19,659 | 1,169.95 | 5.48^#^ | 4.79-6.24 |
| Age at diagnosis, years |  |  |  |  |  |  |
| 0-19 | 391 | 7 | 2,820 | 248.24 | 157.50^#^ | 63.32-324.52 |
| 20-39 | 790 | 26 | 4,635 | 560.91 | 57.77^#^ | 37.74-84.65 |
| 40-59 | 5,503 | 209 | 29,538 | 707.56 | 17.47^#^ | 15.18-20.00 |
| 60-79 | 27,203 | 863 | 99,441 | 867.85 | 5.26^#^ | 4.92-5.63 |
| ≥ 80 | 19,766 | 698 | 46,186 | 1,511.27 | 2.60^#^ | 2.41-2.80 |
| Time since diagnosis, months |  |  |  |  |  |  |
| < 3 | 53,653 | 287 | 12,477.05 | 2,300.22 | 9.20^#^ | 8.17-10.33 |
| 3-5 | 47,702 | 167 | 11,154.33 | 1,497.18 | 6.08^#^ | 5.19-7.08 |
| 6-11 | 43,024 | 270 | 19,476.65 | 1,386.28 | 5.66^#^ | 5.00-6.37 |
| 12-35 | 36,300 | 466 | 54,204.66 | 859.70 | 3.47^#^ | 3.16-3.80 |
| 36-59 | 20,638 | 270 | 32,695.39 | 825.80 | 3.31^#^ | 2.93-3.73 |
| 60-119 | 12,935 | 263 | 39,122.97 | 672.24 | 2.81^#^ | 2.48-3.170 |
| 120-179 | 4,242 | 70 | 11,477.66 | 609.88 | 2.81^#^ | 2.19-3.54 |
| ≥180 | 1,012 | 10 | 2,012 | 497.05 | 2.81^#^ | 1.35-5.170 |
| Gender |  |  |  |  |  |  |
| Female | 23,775 | 777 | 88,780 | 875.20 | 3.94^#^ | 3.67-4.23 |
| Male | 29,878 | 1,026 | 93,841 | 1,093.34 | 4.15^#^ | 3.90-4.41 |
| Race |  |  |  |  |  |  |
| NHW | 40,581 | 1,305 | 135,250 | 964.88 | 3.75^#^ | 3.55-3.96 |
| NHB | 4,078 | 178 | 16,282 | 1,093.22 | 4.92^#^ | 4.23-5.70 |
| Other | 8,994 | 320 | 31,088 | 1,029.32 | 5.26^#^ | 4.70-5.87 |
| Marital status |  |  |  |  |  |  |
| Married | 26,940 | 832 | 94,660 | 878.93 | 3.92^#^ | 3.65-4.19 |
| Single | 5,697 | 209 | 20,949 | 997.66 | 7.18^#^ | 6.24-8.22 |
| D/S/W | 16,200 | 596 | 46,708 | 1,276.03 | 3.89^#^ | 3.58-4.21 |
| Other | 4,816 | 166 | 20,304 | 817.58 | 3.35^#^ | 2.86-3.90 |
| Chemotherapy |  |  |  |  |  |  |
| No/Unknown | 41,934 | 150 | 151,559 | 98.97 | 3.77^#^ | 3.58-3.97 |
| Yes | 11,719 | 302 | 31,062 | 972.26 | 6.49^#^ | 5.77-7.26 |
| Types of infectious disease |  |  |  |  |  |  |
| Septicemia | 53,653 | 656 | 182,621 | 359.21 | 4.99^#^ | 4.62-5.39 |
| Other infections and parasitic  diseases including HIV | 53,653 | 406 | 182,621 | 222.32 | 6.65^#^ | 6.02-7.33 |
| Pneumonia and influenza | 53,653 | 723 | 182,621 | 395.90 | 2.89^#^ | 2.69-3.11 |

IDM, infectious diseases mortality; SMRs, standardized mortality ratios; NHW, non-Hispanic white; NHB, non-Hispanic black; D/S/W, separated/divorced/widowed; # means *P* < 0.05.

Table S8 Incidence and SMRs of infectious diseases mortality in patients with myeloproliferative neoplasms by demographic and clinical characteristics

| Variable | Patients with  hematologic  malignancies No. | IDM  No. | Person-years | IDM per 100,000 Person-years | SMRs | 95% CI |
| --- | --- | --- | --- | --- | --- | --- |
| Period of diagnosis, years |  |  |  |  |  |  |
| 2000-2002 | 3,418 | 39 | 11,770 | 331.35 | 2.20^#^ | 1.56-3.00 |
| 2003-2005 | 5,827 | 60 | 22,055 | 272.04 | 1.85^#^ | 1.41-2.37 |
| 2006-2008 | 6,586 | 88 | 25,125 | 350.25 | 2.52^#^ | 2.02-3.11 |
| 2009-2011 | 6,074 | 66 | 23,770 | 277.66 | 2.02^#^ | 1.56-2.57 |
| 2012-2014 | 6,827 | 57 | 26,593 | 214.34 | 1.68^#^ | 1.27-2.18 |
| 2015-2017 | 7,484 | 74 | 29,101 | 254.29 | 2.13^#^ | 1.67-2.67 |
| Age at diagnosis, years |  |  |  |  |  |  |
| 0-19 | 389 | 0 | 3,327 | - | - | - |
| 20-39 | 3,222 | 8 | 26,163 | 30.58 | 3.25^#^ | 1.40-6.40 |
| 40-59 | 12,493 | 124 | 101,362 | 122.33 | 3.12^#^ | 2.60-3.72 |
| 60-79 | 20,433 | 406 | 122,692 | 330.91 | 2.25^#^ | 2.03-2.48 |
| ≥ 80 | 7,110 | 255 | 26,771 | 952.52 | 1.71^#^ | 1.50-1.93 |
| Time since diagnosis, months |  |  |  |  |  |  |
| < 3 | 43,647 | 49 | 10,620 | 461.39 | 3.54^#^ | 2.62-4.67 |
| 3-5 | 41,825 | 31 | 10,259 | 302.19 | 2.32^#^ | 1.58-3.29 |
| 6-11 | 40,442 | 40 | 19,631 | 203.76 | 1.56^#^ | 1.12-2.13 |
| 12-35 | 38,241 | 187 | 67,729 | 276.10 | 2.08^#^ | 1.79-2.40 |
| 36-59 | 29,755 | 144 | 52,078 | 276.51 | 2.05^#^ | 1.73-2.42 |
| 60-119 | 22,654 | 211 | 79,273 | 266.17 | 1.98^#^ | 1.72-2.27 |
| 120-179 | 10,440 | 106 | 33,337 | 317.96 | 2.43^#^ | 1.99-2.94 |
| ≥180 | 3,463 | 25 | 7,388 | 338.38 | 2.66^#^ | 1.72-3.93 |
| Gender |  |  |  |  |  |  |
| Female | 22,399 | 367 | 143,892 | 255.05 | 1.87^#^ | 1.68-2.07 |
| Male | 21,248 | 426 | 136,423 | 312.26 | 2.42^#^ | 2.20-2.66 |
| Race |  |  |  |  |  |  |
| NHW | 32,126 | 617 | 206,188 | 299.24 | 2.11^#^ | 1.94-2.28 |
| NHB | 4,153 | 67 | 25,165 | 266.24 | 2.05^#^ | 1.59-2.60 |
| Other | 7,368 | 109 | 48,961 | 222.62 | 2.35^#^ | 1.93-2.83 |
| Marital status |  |  |  |  |  |  |
| Married | 22,197 | 347 | 149,565 | 232.01 | 1.92^#^ | 1.73-2.14 |
| Single | 6,538 | 96 | 43,051 | 222.99 | 3.08^#^ | 2.50-3.77 |
| D/S/W | 9,791 | 257 | 55,069 | 466.68 | 2.16^#^ | 1.91-2.44 |
| Other | 5,121 | 93 | 32,629 | 285.02 | 2.21^#^ | 1.79-2.71 |
| Chemotherapy |  |  |  |  |  |  |
| No/Unknown | 26,740 | 497 | 179,862 | 276.32 | 2.36^#^ | 2.16-2.58 |
| Yes | 16,907 | 296 | 100,453 | 294.67 | 1.83^#^ | 1.62-2.05 |
| Types of infectious disease |  |  |  |  |  |  |
| Septicemia | 43,647 | 292 | 280,315 | 104.17 | 2.52^#^ | 2.24-2.83 |
| Other infections and parasitic  diseases including HIV | 43,647 | 149 | 280,315 | 53.15 | 2.34^#^ | 1.98-2.74 |
| Pneumonia and influenza | 43,647 | 345 | 280,315 | 123.08 | 1.81^#^ | 1.62-2.01 |

IDM, infectious diseases mortality; SMRs, standardized mortality ratios; NHW, non-Hispanic white; NHB, non-Hispanic black; D/S/W, separated/divorced/widowed; # means *P* < 0.05.

Table S9 Incidence and SMRs of infectious diseases mortality in patients with plasma cell myeloma by demographic and clinical characteristics

| Variable | Patients with  hematologic  malignancies No. | IDM  No. | Person-years | IDM per 100,000 Person-years | SMRs | 95% CI |
| --- | --- | --- | --- | --- | --- | --- |
| Period of diagnosis, years |  |  |  |  |  |  |
| 2000-2002 | 10,234 | 121 | 25,408 | 476.23 | 2.90^#^ | 2.4-3.46 |
| 2003-2005 | 11,008 | 154 | 29,130 | 528.66 | 3.45^#^ | 2.93-4.04 |
| 2006-2008 | 11,494 | 156 | 32,172 | 484.89 | 3.51^#^ | 2.98-4.11 |
| 2009-2011 | 13,22 | 199 | 38,934 | 511.13 | 3.85^#^ | 3.33-4.42 |
| 2012-2014 | 14,374 | 198 | 43,707 | 453.02 | 3.52^#^ | 3.05-4.04 |
| 2015-2017 | 15,955 | 234 | 49,584 | 471.93 | 3.92^#^ | 3.43-4.45 |
| Age at diagnosis, years |  |  |  |  |  |  |
| 0-19 | 25 | 0 | 188 | - | - | - |
| 20-39 | 1,507 | 18 | 10,038 | 179.33 | 11.77^#^ | 6.98-18.61 |
| 40-59 | 23,907 | 379 | 129,753 | 292.09 | 6.57^#^ | 5.92-7.26 |
| 60-79 | 51,071 | 854 | 190,696 | 447.83 | 3.34^#^ | 3.12-3.57 |
| ≥ 80 | 15,968 | 343 | 31,810 | 1,078.29 | 1.99^#^ | 1.79-2.22 |
| Time since diagnosis, months |  |  |  |  |  |  |
| < 3 | 92,478 | 249 | 21,467 | 1,159.92 | 8.08^#^ | 7.10-9.14 |
| 3-5 | 82,120 | 164 | 19,435 | 843.85 | 6.16^#^ | 5.25-7.18 |
| 6-11 | 75,575 | 150 | 35,277 | 425.20 | 3.17^#^ | 2.68-3.72 |
| 12-35 | 66,978 | 408 | 107,270 | 380.35 | 2.85^#^ | 2.58-3.14 |
| 36-59 | 43,006 | 238 | 69,039 | 344.73 | 2.61^#^ | 2.29-2.96 |
| 60-119 | 27,446 | 290 | 81,055 | 357.78 | 2.69^#^ | 2.39-3.01 |
| 120-179 | 8,516 | 79 | 23,881 | 330.80 | 2.40^#^ | 1.90-3.00 |
| ≥180 | 2,210 | 16 | 5,060 | 316.22 | 2.21^#^ | 1.26-3.58 |
| Gender |  |  |  |  |  |  |
| Female | 41,889 | 605 | 164,916 | 366.85 | 2.94^#^ | 2.71-3.18 |
| Male | 50,589 | 989 | 197,569 | 500.58 | 3.52^#^ | 3.30-3.75 |
| Race |  |  |  |  |  |  |
| NHW | 57,059 | 912 | 224,996 | 405.34 | 2.92^#^ | 2.74-3.12 |
| NHB | 17,638 | 400 | 69,738 | 573.58 | 3.79^#^ | 3.43-4.18 |
| Other | 17,781 | 282 | 67,751 | 416.23 | 4.06^#^ | 3.60-4.57 |
| Marital status |  |  |  |  |  |  |
| Married | 51,771 | 764 | 220,082 | 347.14 | 2.84^#^ | 2.64-3.05 |
| Single | 12,453 | 282 | 46,420 | 607.50 | 6.37^#^ | 5.65-7.16 |
| D/S/W | 22,030 | 424 | 69,688 | 608.43 | 3.16^#^ | 2.86-3.47 |
| Other | 6,224 | 124 | 26,295 | 471.58 | 3.14^#^ | 2.61-3.74 |
| Chemotherapy |  |  |  |  |  |  |
| No/Unknown | 38,338 | 766 | 146,740 | 522.01 | 3.14^#^ | 2.92-3.37 |
| Yes | 54,140 | 828 | 215,745 | 383.79 | 3.40^#^ | 3.17-3.64 |
| Types of infectious disease |  |  |  |  |  |  |
| Septicemia | 92,478 | 492 | 362,485 | 135.73 | 3.07^#^ | 2.8-3.35 |
| Other infections and parasitic  diseases including HIV | 92,478 | 425 | 362,485 | 117.25 | 4.76^#^ | 4.32-5.23 |
| Pneumonia and influenza | 92,478 | 672 | 362,485 | 185.39 | 2.86^#^ | 2.65-3.09 |

IDM, infectious diseases mortality; SMRs, standardized mortality ratios; NHW, non-Hispanic white; NHB, non-Hispanic black; D/S/W, separated/divorced/widowed; # means *P* < 0.05.
